# Supplementary material for: MetaRibo-Seq measures translation in microbiomes
Source: Nat Commun. 2020 Jun 29;11:3268. doi: 10.1038/s41467-020-17081-z (PMC7324362; doi:10.1038/s41467-020-17081-z)
Supplement: Supplementary file 10 — Supplementary Data 7 [file 41467_2020_17081_MOESM10_ESM.zip › File2/Confidence_High_Taxonomy/367387_out.krona.html]

Javascript must be enabled to view this page.

members
magnitude
magnitudeUnassigned
count
unassigned
taxon
rank

367387\_out

201

superkingdom
201
2

1239
201
phylum

class
201
186801

8

SRS014979\_contig\_number\_contig-100\_3403.3404SRS024549\_contig\_number\_contig-100\_1179.49658SRS048060\_contig\_number\_contig-100\_33213.33214SRS051882\_contig\_number\_contig-100\_2456.138992SRS052697\_contig\_number\_contig-100\_2235.44512SRS076804\_contig\_number\_contig-100\_36027.36028SRS1041037\_contig\_number\_contig-100\_10547.71408SRS144714\_contig\_number\_contig-100\_11672.11673
186802
order
201

541000
family
17

1
species

SRS011302\_contig\_number\_19517
1898205

1263
16
genus

165186

SRS013687\_contig\_number\_33471SRS015217\_contig\_number\_contig-100\_286.181890SRS015854\_contig\_number\_contig-100\_6792.74948SRS022524\_contig\_number\_14099SRS042966\_contig\_number\_2646SRS051882\_contig\_number\_22717SRS064276\_contig\_number\_42816SRS1041095\_contig\_number\_6199SRS1041146\_contig\_number\_contig-100\_21.18500SRS1041147\_contig\_number\_4881SRS1054928\_contig\_number\_6317SRS143780\_contig\_number\_contig-100\_15979.141355SRS144537\_contig\_number\_45052SRS147022\_contig\_number\_12517SRS148970\_contig\_number\_2579
15
species

species
1

SRS142503\_contig\_number\_40217
2293178

family
2
186806

1730
2
genus


SRS018623\_contig\_number\_3545SRS019496\_contig\_number\_14708
142586
species
2

1

SRS043768\_contig\_number\_contig-100\_44238.44239
31979
45
family


SRS021948\_contig\_number\_14630
2305246
1
species

7

SRS014459\_contig\_number\_contig-100\_2858.238900SRS014613\_contig\_number\_11073SRS063040\_contig\_number\_contig-100\_13625.192750SRS1055083\_contig\_number\_contig-100\_17825.53150SRS147766\_contig\_number\_16613SRS149879\_contig\_number\_contig-100\_1576.160747SRS893253\_contig\_number\_contig-100\_14942.14943
1485
genus
43

species
1

SRS148253\_contig\_number\_14338
2293031

1
species
59620

SRS893378\_contig\_number\_21178


SRS014287\_contig\_number\_18757
2293002
species
1


SRS015486\_contig\_number\_1115SRS018351\_contig\_number\_contig-100\_52.108903SRS078419\_contig\_number\_contig-100\_123.227170SRS140513\_contig\_number\_15559
665940
4
species


SRS014736\_contig\_number\_9336SRS017521\_contig\_number\_31110SRS019267\_contig\_number\_contig-100\_1796.1796SRS019685\_contig\_number\_26969SRS023715\_contig\_number\_11031SRS050422\_contig\_number\_15138SRS064973\_contig\_number\_386SRS078242\_contig\_number\_contig-100\_440.165147SRS146888\_contig\_number\_7703
1262842
9
species

species
1
2293023

SRS063518\_contig\_number\_39966

1
species
2305242

SRS012849\_contig\_number\_28998

2293051

SRS013521\_contig\_number\_14729SRS015264\_contig\_number\_17781SRS019161\_contig\_number\_6510SRS049995\_contig\_number\_contig-100\_2754.211641SRS050299\_contig\_number\_7430SRS051031\_contig\_number\_21231SRS056519\_contig\_number\_14542SRS063127\_contig\_number\_contig-100\_4202.4203SRS063518\_contig\_number\_39158SRS077086\_contig\_number\_11245SRS104311\_contig\_number\_45507SRS143722\_contig\_number\_16107SRS143722\_contig\_number\_contig-100\_6644.82677SRS148253\_contig\_number\_contig-100\_1775.94629SRS893270\_contig\_number\_10565SRS893295\_contig\_number\_contig-100\_2369.80374SRS893383\_contig\_number\_34539
17
species


SRS021484\_contig\_number\_32163
1262824
1
species

129
family

SRS013951\_contig\_number\_contig-100\_30845.129107SRS013965\_contig\_number\_34098SRS014459\_contig\_number\_42318SRS015065\_contig\_number\_39880SRS015431\_contig\_number\_77111SRS016495\_contig\_number\_4999SRS018427\_contig\_number\_32698SRS019068\_contig\_number\_contig-100\_1691.642271SRS019787\_contig\_number\_contig-100\_11511.43879SRS023526\_contig\_number\_contig-100\_2637.186380SRS024132\_contig\_number\_34271SRS043001\_contig\_number\_8299SRS053335\_contig\_number\_17565SRS075341\_contig\_number\_contig-100\_3826.137899SRS098881\_contig\_number\_contig-100\_13437.62968SRS1041037\_contig\_number\_contig-100\_7949.81721SRS144297\_contig\_number\_contig-100\_6108.83259SRS146812\_contig\_number\_contig-100\_2852.287541SRS148511\_contig\_number\_contig-100\_1734.135487SRS893259\_contig\_number\_contig-100\_1469.55300SRS893358\_contig\_number\_contig-100\_22699.22700
21
186803

genus
1
830


SRS104693\_contig\_number\_13027
45851
species
1

189330
genus
14

species
12
1263073

SRS012969\_contig\_number\_contig-100\_300.237747SRS018984\_contig\_number\_17104SRS043768\_contig\_number\_15326SRS048870\_contig\_number\_17164SRS049773\_contig\_number\_contig-100\_13.210054SRS052027\_contig\_number\_5237SRS077552\_contig\_number\_29949SRS098644\_contig\_number\_contig-100\_19795.168237SRS1041039\_contig\_number\_24139SRS142599\_contig\_number\_30017SRS148424\_contig\_number\_32404SRS893292\_contig\_number\_23

species
2
88431

SRS077849\_contig\_number\_32027SRS1041130\_contig\_number\_contig-100\_180.50120

841
genus
6


SRS065504\_contig\_number\_23070SRS077454\_contig\_number\_19608SRS098061\_contig\_number\_162SRS144183\_contig\_number\_30126
166486
species
4

species
1
1262940

SRS017191\_contig\_number\_11414


SRS142542\_contig\_number\_25698
2293144
species
1

572511
genus
43

5
species
1322

SRS017916\_contig\_number\_4502SRS1055022\_contig\_number\_contig-100\_47.140790SRS893288\_contig\_number\_contig-100\_62.36645SRS893295\_contig\_number\_13637SRS893300\_contig\_number\_5453

40520

SRS011084\_contig\_number\_38011SRS014235\_contig\_number\_40469SRS014923\_contig\_number\_46898SRS015578\_contig\_number\_37407SRS015890\_contig\_number\_15609SRS016095\_contig\_number\_26480SRS016267\_contig\_number\_839SRS018575\_contig\_number\_3035SRS020328\_contig\_number\_16136SRS043841\_contig\_number\_11179SRS044535\_contig\_number\_contig-100\_499.142600SRS045195\_contig\_number\_2604SRS045713\_contig\_number\_contig-100\_2480.100650SRS048981\_contig\_number\_6793SRS049959\_contig\_number\_51680SRS053356\_contig\_number\_47856SRS054956\_contig\_number\_15730SRS058070\_contig\_number\_contig-100\_15.184700SRS062654\_contig\_number\_26818SRS062701\_contig\_number\_7860SRS063985\_contig\_number\_16490SRS074964\_contig\_number\_5714SRS097889\_contig\_number\_40713SRS098644\_contig\_number\_31390SRS104084\_contig\_number\_13091SRS1041112\_contig\_number\_6555SRS1041142\_contig\_number\_contig-100\_1617.52137SRS104400\_contig\_number\_58057SRS142923\_contig\_number\_22337SRS143342\_contig\_number\_14430SRS143598\_contig\_number\_21722SRS144362\_contig\_number\_39548SRS146764\_contig\_number\_34029SRS147614\_contig\_number\_10694SRS147919\_contig\_number\_21692SRS148511\_contig\_number\_21855SRS148874\_contig\_number\_16268SRS149181\_contig\_number\_17829
38
species

species
1
2292271

SRS055017\_contig\_number\_contig-100\_449.201629

species
2
2109691

SRS015663\_contig\_number\_34470SRS142503\_contig\_number\_59322

species
3

SRS077730\_contig\_number\_18687SRS1041143\_contig\_number\_3748SRS147377\_contig\_number\_8374
2002841

39491

SRS014534\_contig\_number\_contig-100\_7.14092SRS014948\_contig\_number\_16168SRS016954\_contig\_number\_contig-100\_12301.12301SRS017433\_contig\_number\_contig-100\_8659.78015SRS019178\_contig\_number\_4811SRS019910\_contig\_number\_contig-100\_7340.30503SRS021219\_contig\_number\_10154SRS022137\_contig\_number\_20864SRS024009\_contig\_number\_10150SRS024625\_contig\_number\_17164SRS045004\_contig\_number\_11510SRS045244\_contig\_number\_3406SRS046717\_contig\_number\_2965SRS048164\_contig\_number\_28021SRS054352\_contig\_number\_contig-100\_8023.8023SRS054590\_contig\_number\_contig-100\_537.28419SRS062701\_contig\_number\_contig-100\_343.67716SRS077127\_contig\_number\_contig-100\_332.102275SRS098571\_contig\_number\_67355SRS1054691\_contig\_number\_1941SRS142712\_contig\_number\_contig-100\_61.47202SRS143372\_contig\_number\_10861SRS893231\_contig\_number\_contig-100\_15.49623SRS893366\_contig\_number\_17124
species
24

1506553
genus
10

species
3
208479

SRS043667\_contig\_number\_6559SRS056273\_contig\_number\_24186SRS1055076\_contig\_number\_17425


SRS042628\_contig\_number\_36838SRS055982\_contig\_number\_contig-100\_117.119488SRS064232\_contig\_number\_contig-100\_18981.18982SRS1041091\_contig\_number\_15734SRS1041138\_contig\_number\_contig-100\_12106.12107SRS143991\_contig\_number\_23189SRS144135\_contig\_number\_32401
1531
species
7


SRS015782\_contig\_number\_contig-100\_3178.225131SRS016897\_contig\_number\_contig-100\_1458.1459SRS050925\_contig\_number\_contig-100\_1901.194702SRS148159\_contig\_number\_58112
1952029
4
species
